# Multi-step down-regulation of the secretory pathway in mitosis: A fresh perspective on protein trafficking

**DOI:** 10.1002/bies.201200144

**Published:** 2013-03-12

**Authors:** Foong May Yeong

**Affiliations:** Department of Biochemistry, Yong Loo Lin School of Medicine, National University of SingaporeSingapore, Singapore

**Keywords:** CDK1, cell division cycle, endoplasmic reticulum, Golgi, mitosis, protein trafficking

## Abstract

The secretory pathway delivers proteins synthesized at the rough endoplasmic reticulum (RER) to various subcellular locations via the Golgi apparatus. Currently, efforts are focused on understanding the molecular machineries driving individual processes at the RER and Golgi that package, modify and transport proteins. However, studies are routinely performed using non-dividing cells. This obscures the critical issue of how the secretory pathway is affected by cell division. Indeed, several studies have indicated that protein trafficking is down-regulated during mitosis. Moreover, the RER and Golgi apparatus exhibit gross reorganization in mitosis. Here I provide a relatively neglected perspective of how the mitotic cyclin-dependent kinase (CDK1) could regulate various stages of the secretory pathway. I highlight several aspects of the mitotic control of protein trafficking that remain unresolved and suggest that further studies on how the mitotic CDK1 influences the secretory pathway are necessary to obtain a deeper understanding of protein transport.

## Introduction

**Figure 1:**
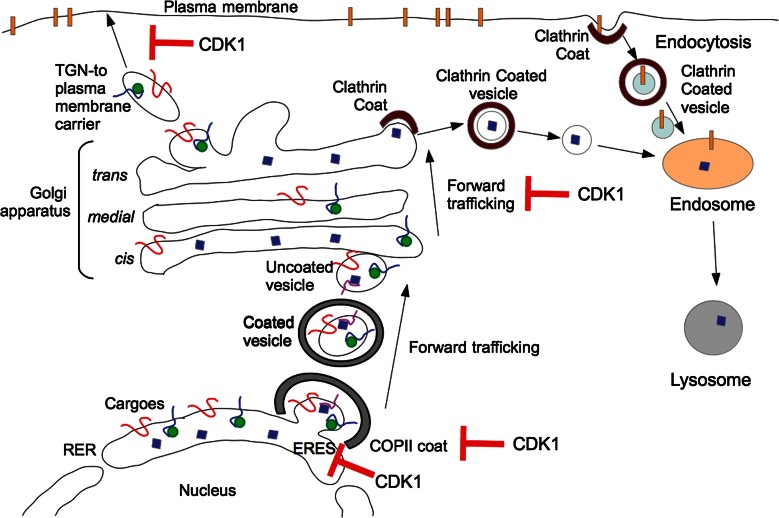
A simplified overview of the secretory and endocytosis pathways. Cargoes at the rough endoplasmic reticulum (RER) could be membrane proteins (red wavy lines) or luminal proteins (green circles) that associate with membrane receptors (blue curved line). The cargoes are recognized by the coatomer protein complex II (COPII) coat protein, Sec24 (see text for details). The COPII (gray) forces the curvature of the RER, leading to the budding off of the RER membrane with the secretory cargoes. Upon release from the RER, the coated COPII vesicle undergoes uncoating and the uncoated vesicle fuses with the *cis*-Golgi (see text for details). The cargoes are delivered to the Golgi apparatus, where they are modified post-translationally. From the *trans*-Golgi network (TGN), the TGN-to-plasma membrane transport carriers deliver the cargoes to the plasma membrane. The membrane protein cargoes become plasma membrane proteins, while the soluble proteins are secreted out of the cell. Lysosomal proteins (blue squares) are sorted at the TGN into clathrin-coated vesicles (maroon) that transport them to the endosome (beige oval). From the endosome, the lysosomal proteins are delivered to the lysosomes (light gray circle). Several steps in the secretory pathway that are likely to be inhibited by cyclin-dependent kinase 1 (CDK1) are shown (see text for details). Plasma membrane proteins (orange rectangles) are internalized by clathrin-coated vesicles in a process known as endocytosis. These membrane proteins (and other extracellular proteins) taken up the cell are brought to the endosome, where they are processed in various ways for further use by the cell.

The timely localization of intracellular proteins is required for the survival of cells. A key pathway needed for the proper subcellular localization of proteins is the secretory pathway that transports proteins from the rough endoplasmic reticulum (RER) via the Golgi to the lysosome, plasma membrane, or the exterior of the cell 1–3 (Fig. 1). The trafficking of cargoes is also required for maintaining the polarity of cells, which is important in specific tissues 4, 5.

Whereas the secretory pathway functions in the forward trafficking of proteins, endocytosis pathways also exist that contribute to the internalization of plasma membrane and plasma membrane proteins, and in the uptake of nutrients 6, 7 (Fig. 1). Endocytosed proteins or membranes are transported to the endosome, an organelle needed for processes such as the sorting, recycling, and degradation of internalized cargoes 8. For instance, endocytosed cell surface receptors could be recycled back to the plasma membrane or degraded, depending upon the signaling requirements of the cell. The regulation of endocytosis is complex and appears to be inhibited during early mitosis, although specialized endocytosis pathways could occur during late mitosis 9, 10. While endocytosis and the endosomal pathway play important roles in intracellular protein trafficking, these topics are beyond the scope of this review, which is focused on the secretory pathway, and readers are referred to the references cited.

Protein trafficking through the secretory pathway is no less complicated than endocytosis and occurs in a highly regulated manner 11. While we have an appreciable understanding of the molecular events occurring during protein trafficking, our knowledge about the secretory pathway has come mostly from studies using non-dividing cells. It is important to note that cells, when exposed to appropriate mitogenic signals, undergo at least several rounds of division throughout their life cycle. As such, a relatively under-appreciated question pertinent to our continued efforts in studying the secretory pathway relates to how cells regulate protein trafficking during cell division.

**Figure 2:**
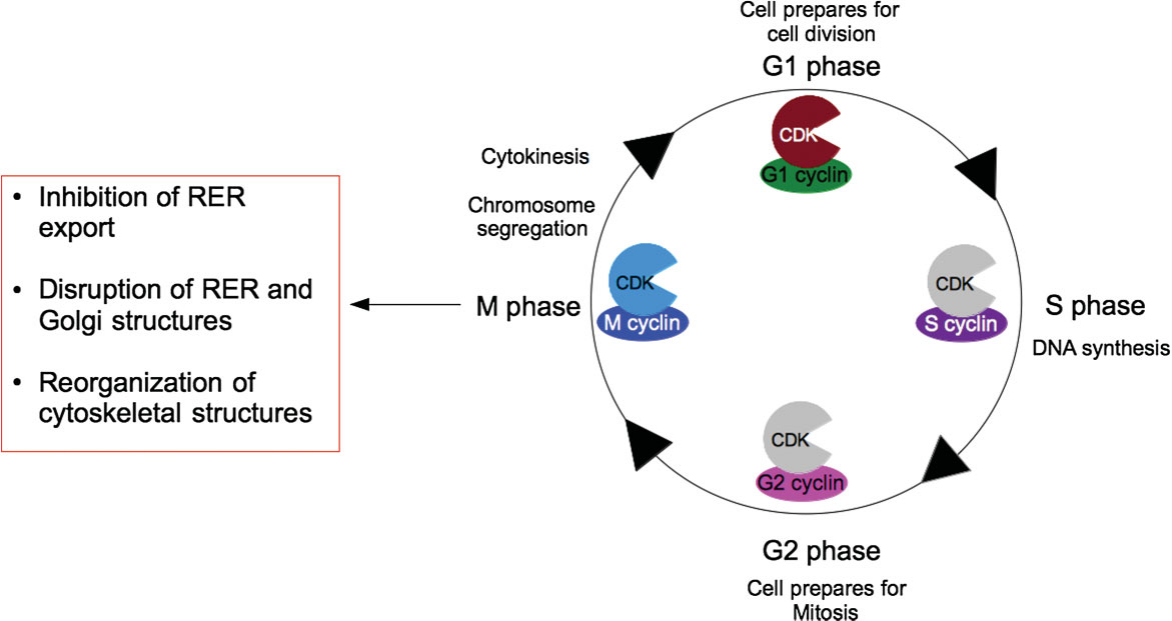
A representation of a typical eukaryotic cell division cycle. The four major phases of the cell division cycle, G1 (gap) phase, S (synthesis) phase, G2 and M (mitosis) phases, are shown. Different cyclins and CDKs function during specific cell cycle phases to drive phase-specific events. Highlighted in the figure are the key processes of the secretory pathway that are inhibited during M phase (see text for details).

Cell division itself is a complex process with the main regulators being the cyclin-dependent kinases (CDKs) and their associated cyclins. Essentially, at each cell cycle phase, specific cyclins bind to their respective CDKs to form cyclin-CDK complexes that promote phase-specific events 12, 13 (Fig. 2). For instance, in mammalian cells at gap phase 1 (G1), the presence of cyclins such as cyclin D, which associates with CDK4 and CDK6, is needed for the execution of G1 events. Other cyclins, when synthesized at subsequent stages, function together with yet other CDKs for the execution of later events of the cell cycle such as DNA synthesis and mitosis.

Given the scant attention paid to studying the secretory pathway with respect to the cell division cycle, I describe here several key findings indicating that protein trafficking is down-regulated in mitosis. I highlight interesting but relatively unexplored data showing functional interactions between various components of the protein trafficking machinery and the major kinase in mitosis, the mitotic cyclin-CDK1 complex (Fig. 1). I end with my perspectives on unresolved issues surrounding the mechanisms underlying the regulation of the secretory pathway by the CDK1 complex. I suggest that, by examining protein trafficking in the context of mitosis, additional levels of control of the secretory pathway could be revealed that are not obvious from studies using non-dividing cells. This would not only improve our understanding of the secretory pathway but also reveal details of the functional interaction between the mitotic CDK1 and protein trafficking that ensures the survival of a cell during its life cycle.

## Overview of protein export from the RER

### Coatomer protein complex II coats recruited to the RER membrane help generate vesicles and select for cargoes

As protein export from the RER has been reviewed recently 2, 3, 14–16, here I summarize only the major events of the process. Protein export is initiated when the membrane protein Sec12, a guanine nucleotide exchange factor (GEF), catalyzes the GDP-to-GTP exchange in the cytoplasmic Sar1p-GDP. This leads to the extension of the N-terminal amphipathic α-helix in Sar1-GTP that anchors it in the RER membrane. Sar1-GTP recruits coatomer protein complex II (COPII) proteins Sec23/Sec24 to the RER membrane to form the “pre-budding complex”. Additional COPII coat proteins Sec13 and Sec31 subsequently associate with Sec23/Sec24 to assemble an outer coat on the pre-budding complex (Fig. 1, gray coat).

The COPII coat forces the curvature of the RER membrane (Fig. 1, gray coat) 2, 3, resulting eventually in the budding off of a transport vesicle encased by the COPII coat. COPII vesicles bud at exit sites (ERES) or transitional ER (tER; Fig. 1) 17 on the RER membrane that are devoid of ribosomes 18. More importantly, secretory cargoes are likely incorporated into the COPII vesicles at the ERES. Numerous ERES are found on the RER of mammalian cells and they are observed as relatively stable and immobile structures 19, 20. Different types of ERES exist depending upon the nature of the cargoes that are recruited to the ERES 21.

In mammalian cells, a peripheral RER membrane protein Sec16A localized at the ERES 22, 23 is needed at a stoichiometric level for the proper assembly of ERES 22. Sec16A interacts with COPII components such as Sec13 and Sec12 24–26 and likely organizes the COPII proteins at the ERES. Consistent with this idea, the budding yeast Sec16 ortholog 27 is able to recruit the COPII coat proteins Sar1-Sec23p-Sec24p on to liposomes 28.

COPII coat proteins help select cargoes for incorporation into COPII vesicles 15. Sec24 and its isoforms each have multiple cargo recognition sites that bind to membrane cargoes. These sites span the RER membrane with one or more regions exposed to the cytoplasm (Fig. 1, red wavy line). Soluble cargoes in the RER lumen (Fig. 1, green circles) rely on association with RER-membrane-spanning proteins that serve as receptors or adaptors (Fig. 1, blue curved line) interacting with Sec24 on the cytoplasmic side of the ERES.

### Various protein complexes are needed to tether COPII vesicles to the destination membrane

Upon budding from the RER, the COPII-coated vesicles either uncoat fully or partially 21. The vesicles travel along microtubules (see below) from the RER towards the *cis*-Golgi or an ER-to-Golgi intermediate compartment (ERGIC) in mammalian cells. Rab GTPases are recruited to the COPII vesicles and upon activation in the presence of GEFs, exchange GDP to GTP 29. Through association with specific Rab effectors, Rab-GTP facilitates docking of vesicles onto the ERGIC 30.

Various other proteins known as tethering factors are required for establishing the initial association between vesicles and the destination membrane 21, 31. These factors are classified either as coiled-coil tethers or multi-subunit tethering complexes and have been implicated in different stages of the protein trafficking pathway such as RER-to-Golgi and intra-Golgi transport. For instance, p115 is a coiled-coil protein functioning as a Rab effector required for tethering of vesicles in RER-to-Golgi trafficking. A multi-subunit tethering complex involved in RER-to-Golgi transport is the transport protein particle (TRAPP1) found at the Golgi. TRAPP1 tethers COPII vesicles to the Golgi possibly via its association with Sec23 29, 32. Furthermore, Bet3, a subunit of TRAPP1, functions as the GEF that promotes GDP-to-GTP switch in Rab1, thereby enabling vesicles to dock at the Golgi membrane through Rab1-GTP-Rab effector association.

Subsequent to the actions of Rab GTPases and tethering proteins, soluble *N*-ethylmaleimide-sensitive fusion protein (NSF)-attachment protein receptors on the vesicle membrane (vSNAREs) and target (t)SNAREs on the ERGIC membrane bring the membranes closer to each other so as to enable membrane fusion and delivery of the vesicle contents to the destination 30, 33. Depending on the organism, the uncoated vesicles could fuse to the *cis*-Golgi (Fig. 1) or undergo homotypic fusion to form ERGICs or vesicular tubular clusters (VTCs), which then mature into or are then transported to the *cis*-Golgi (e.g. in mammalian cells) 1, 21, 30.

### Anterograde and retrograde transport of cargoes occur in the Golgi apparatus

Several different models of intra-Golgi transport, such as the progression/maturation model (and variations of that), have been suggested 33, 34 to explain how cargoes traverse the *cis*-, *medial*-, and *trans*-Golgi. The proposed progression/maturation models are based on the idea that cargoes move through the Golgi stack in a non-vesicular manner. A pathway known as the retrograde pathway, involving the COPI vesicles, retrieves proteins and membranes from the Golgi to the RER and within the Golgi apparatus 1. Presumably the COPI vesicles recycle enzymes and proteins from the medial and *trans*-Golgi to the *cis*-Golgi, leading to the maturation of the *cis*-Golgi. A generally accepted consensus is the post-translational modification of cargoes by glycosylation or disulphide bond formation during passage through the *cis*-, *medial*-, and *trans*-Golgi network (TGN; Fig. 1).

A COPI-independent pathway also functions to recycle proteins and lipids from the Golgi to the RER 35, 36. Proteins transported via the COPI-independent pathway, unlike protein cargoes of the COPI-dependent pathway, do not require the characteristic KDEL recognition sequence and the KDEL receptor in the vesicles. The COPI-independent pathway might be functionally more relevant for recycling of membranes in the secretory pathway.

### Cargoes arriving at the trans-Golgi network are sorted for transport to various subcellular locations

Upon reaching the TGN, specific cargoes are sorted for delivery to different subcellular locations such as the lysosomes, plasma membrane, and endosomes 37, 38. Sorting signals are required to target specific cargoes to the right destinations, although the signals and machinery needed for cargo sorting are not entirely understood.

In mammalian cells, TGN-to-plasma membrane transport carriers ranging from several hundred nanometers to 1.7 µm in length are formed when membranes encompassing cargoes are pulled off from the TGN (Fig. 1) 39. Both actin and microtubules, and their associated motor proteins, promote the budding, elongation, and fission of the TGN membrane to give rise to post-TGN transport carriers 40.

Transport of lysosomal proteins from the TGN goes through the endosomes (Fig. 1, blue squares) and requires transport carriers that are coated with adaptor proteins, GGAs (Golgi-localized, γ-ear-containing, ADP ribosylation factor-binding proteins), and clathrin 8, 37, 38. For TGN-to-plasma membrane trafficking, such as in polarized epithelial cells, distinct vesicular transport carriers convey proteins to the apical, rather than to the basal, membrane 37, 40. Trafficking of basolateral membrane proteins is better understood and is thought to be mediated by membrane carriers coated with adaptor proteins and clathrin. Presently, the coat and adaptor proteins for most transport carriers are unclear, except for those delivering cargoes to the endosomes and basolateral membranes 38, 39.

Transmembrane protein cargoes are incorporated as integral membrane proteins upon fusion of the transport carriers from the TGN with the destination membrane such as the plasma membrane (Fig. 1, red wavy lines) 37. Other types of membrane proteins are delivered to the plasma membrane indirectly via the endosomes. Soluble proteins within the lumen of the transport carriers targeted to the plasma membrane will be secreted out of the cell (Fig. 1, green circles).

### Microtubules and actin are required in vesicular protein transport

Vesicular transport in various stages of the secretory pathway is facilitated by cytoskeletal structures. In mammalian cells, microtubules emanate outwards from the microtubule-organizing center (MTOC) towards the cell cortex 41. This means that vesicles from the cortical RER travel from the microtubule plus-ends towards the minus-ends, where the Golgi apparatus is generally located proximal to the MTOC. This is dependent upon the dynein-dynactin motor protein complex 41, 42. Dynein, a minus-end directed motor, is recruited to the COPII vesicles at the ERES via dynactin that binds to the COPII coat proteins. As dynein is a processive motor, it moves COPII vesicles from the RER on the microtubule tracks towards the Golgi apparatus. Transport carriers that subsequently emerge from the TGN travel to the cell cortex along microtubules with the help of plus-end-directed kinesin motors. Various kinesin motors function differently for the intracellular translocation of the vesicles, although not all of the motors are directly needed for the movement of secretory vesicles.

The transport of vesicular carriers could further depend upon actin filaments and myosin motors. For instance, the movement of specialized transport carriers such as secretory granules (SGs) from the TGN to the cortical region in neuroendocrine PC12 cells requires microtubules and kinesin-1. Upon reaching the cortical region, the SGs rely upon actin filaments and the myosin motor, Myo5a, for delivery to the plasma membrane, resulting eventually in the fusion of the SGs to the plasma membrane and release of the neuropeptides out of the cell 43. Although Myo5a is thought to move SGs along the actin filaments, it has also been suggested that, as a non-processive motor, Myo5a acts only to dock the SGs onto the plasma membrane. Other non-processive myosins such as Myo2 might influence the movement of the vesicles at the cell cortex through their actions in organizing actin filaments.

That microtubules and actin filaments are necessary for protein trafficking has also been reported for the glucose transporter Glut-4 in cells responding to insulin 44. However, the exact roles of the cytoskeleton in Glut-4 transport are still unclear, given that contradictory observations of the requirement for microtubules and actin have been noted. Nevertheless, it is generally thought that microtubule-associated motor proteins facilitate long-range movements of secretory vesicles along microtubules, while actin, together with its motor proteins, mediates short-range movements at the cell cortex.

## Protein trafficking is down-regulated during mitosis

Studies on the secretory pathway have thus far revealed the complex processes needed to ensure the successful stepwise delivery of cargoes from one compartment to the next. As these studies have largely been conducted in non-dividing cells, details pertaining to how the secretory pathway is altered during cell division are unclear. Interestingly, several reports have described data showing the down-regulation of protein trafficking during mitosis. From the data discussed below, it is clear that understanding how protein transport is regulated in mitosis is an important step towards building a more comprehensive model of protein trafficking in the cell.

### Various cargoes are retained in the RER during mitosis

The notion that protein trafficking is negatively regulated during mitosis is supported by studies using the membrane-spanning G protein of the vesicular stomatitis virus (VSV-G) as a protein cargo. In normal rat kidney 45 and Chinese hamster ovary (CHO) cells 46, VSV-G was transported to the plasma membrane in interphase but was retained in the RER when cells entered mitosis. Other membrane proteins, such as the RER-to-Golgi marker ERGIC53/p58 47 and CD8 co-receptor, accumulated in the RER during mitosis 48, hinting at the possibility of disrupted cargo export.

The down-regulation of the secretory pathway in mitosis also extends to soluble proteins, as a truncated VSV-G lacking the transmembrane domain, which behaves like a luminal protein, was found at the RER in mitotic cells 49. Another soluble cargo, the human growth hormone, was secreted out of mitotic CHO cells at a level 10-fold lower than in interphase cells 49. A similar inhibition in mitotic 2H3 rat mast cells was also reported for the regulated secretion of histamine 50.

It is unclear what the mechanism is that underlies the retention of cargoes at the RER in mitosis 45, 46, 49. This is true for exogenously expressed cargoes such as VSV-G and various endogenous cargoes. Intriguingly, de-*O*-glycosylation and phosphorylation of Sec24 have previously been noted in mitotic but not interphase cells 51. This correlates with a loss of recruitment of Sec24 to membranes during mitosis 19, 51–53 and implies that post-translational modifications might affect the ability of COPII coats to associate with the RER. While the evidence suggests that the mitotic phase negatively regulates protein export, there is as yet no direct evidence suggesting that mitotic CDK1 activity reduces the recruitment of COPII coat proteins to the RER and hence perturb cargo export.

### Disruption to RER export could be due to the dismantling of ERES by the mitotic CDK1 activity

The down-regulation of the secretory pathway might stem from a disruption to the initial steps of cargo export at the RER. In interphase CHO and HeLa cells, presumptive ERES are marked by COPII coat proteins such as Sec13 19. Notably, in mitotic cells, Sec13 and other COPII coat proteins were found dispersed to the cytoplasm 51–53. Moreover, the ERGIC-53/p58 cargo receptor showed decreased Sec13 association at the ERES during mitosis 19, indicating that a reduced ability to assemble the COPII coats at the ERES in mitosis contributed to cargo retention at the RER. Interestingly, Sec16A remains associated with the RER membranes during mitosis, possibly at the ERES 54. It was proposed that this enables a quick reassembly of ERES at the end of mitosis to restore the secretory pathway.

Metaphase cells have been shown to exhibit shortened buds protruding at the ERES as compared to those in interphase 51–53. ERES assembly also appears to be perturbed by the mitotic CDK1, since the addition of either mitotic cell extracts or mitotic cyclin-CDK1 complex to streptolysin-O-permeabilized CHO cells led to the disassembly of ERES as judged by the YIP1A marker 55. It would be useful to confirm the findings using additional ERES markers such as Sec16A 22, 23. This is because, although YIP1A recently has been implicated COPII vesicle biogenesis 56, its localization and function at ERES have not been further characterized.

It is unknown what the targets of the mitotic cyclin-CDK1 are that play a role in ERES integrity. Intriguingly, an essential co-factor of Golgi biogenesis, p47, was phosphorylated by the mitotic cyclin-CDK1 57 and overexpression of a non-phosphorylatable form of p47 blocked ERES disassembly in cells 55. However, as p47 is also involved in the assembly of the Golgi apparatus, the implication of this observation is unclear. Nonetheless, the dispersal of COPII components and alterations in ERES structure at the RER due to CDK1 activity could reduce cargo recruitment to COP II vesicle-budding sites for export. Investigating how COPII coat proteins and ERES assembly might be affected by the mitotic CDK1 could uncover details about COPII recruitment to the RER and ERES formation that might not be apparent when studied using interphase cells.

The level of cargoes in the RER has in turn been shown to affect the number of ERES 58, 59. Given that protein synthesis is down-regulated during mitosis to 25–30% of interphase levels 60, it is conceivable that the reduction in protein trafficking could be due to a decline in protein synthesis 61. In this regard, the mitotic CDK1 activity has been found to promote the disassembly of the nucleolus 62 and a reduction in mRNA translation 60, 63, and as such, could indirectly down-regulate the number of ERES.

### Intra-Golgi transport is interrupted during mitosis

Using an in vitro Golgi reconstitution assay that examines the status of glycosylation of VSV-G as an indication of passage through the Golgi 64, it was noted that the inclusion of cyclin A or mitotic extracts from HeLa cells in the in vitro assays inhibited intra-Golgi transport 65, 66. Conversely, the addition of an inactivated temperature-sensitive CDK1 from FT210 cells 65 or the kinase inhibitor staurosporine 65 had no adverse effects on proper VSV-G glycosylation in the in vitro assay. These observations point to the likelihood that CDK1 also acts to prevent intra-Golgi trafficking, albeit via an indirect manner 65.

The effect of the mitotic phase on trafficking through the Golgi apparatus was corroborated by a study that examined the transport of glycosphingolipids (GSLs) from the *cis*- to the *trans*-Golgi 67. The conversion of endogenous ceramide radiolabeled by [^3^H]serine to various intermediates of GSLs through the Golgi apparatus was examined as an indication of transport through the Golgi apparatus. A late intermediate, G_A2_(GalNAc(β1,4)LacCer, was detected in interphase but not mitotic cells. The authors excluded the possibility that the enzyme needed for the synthesis of G_A2_(GalNAc(β1,4)LacCer, UDP-GalNAc:lactosylceramide(βl-4)N-acetylgalactosaminyltransferase (GalNAcT I), was absent or inactive during mitosis, and hence suggested that the absence of the GSL intermediate was indeed due to a block in intra-Golgi transport.

**Figure 3:**
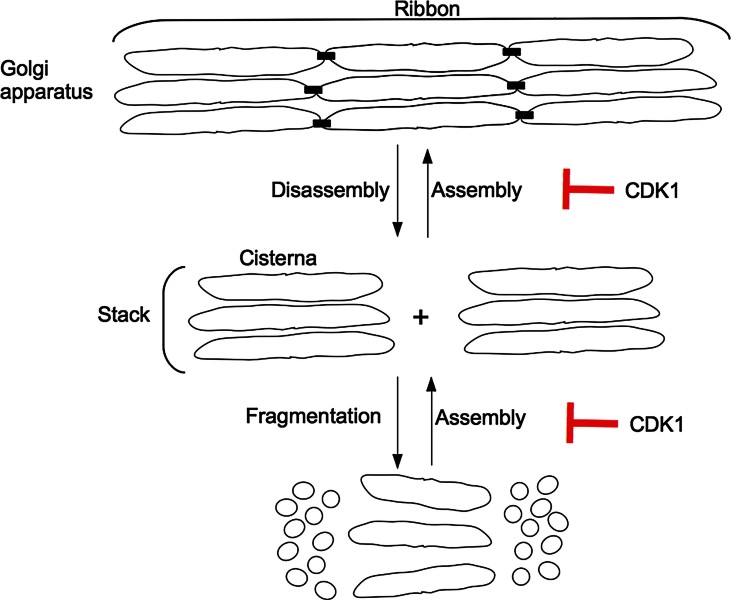
Assembly and disassembly of the Golgi apparatus (see text for details). Three to four Golgi cisternae form a stack (middle panel), which associate with each other laterally to form ribbons (top panel) during assembly. During disassembly, the ribbons are broken down into stacks of cisternae, which might undergo fragmentation leading to the formation of vesicles and breakdown of the Golgi apparatus.

A likely factor contributing to the interruption of intra-Golgi transport could be the fragmentation of the Golgi apparatus during mitosis (Fig. 3) 68–71. The Golgi apparatus is a stack of flattened membrane-bound compartments known as cisternae 72. Typically, 4–11 cisternae form a Golgi stack in mammalian cells (Fig. 3) 73. The cisternae are linked laterally by tubules to form Golgi ribbons that exist during interphase (Fig. 3). In mitosis, the Golgi apparatus undergoes fragmentation that aids Golgi inheritance during cell division (Fig. 3) 68, 74. This appears to be mediated by the mitotic CDK activity, as constitutively active CDK1 and cyclin B1 or cyclin B2, when co-expressed in G1 CHO cells devoid of endogenous mitotic CDK1 complexes, led to the disassembly of the Golgi apparatus 75.

### Is TGN-to-surface transport unaffected during mitosis?

The TGN-to-plasma membrane trafficking depends upon transport carriers such as SGs that carry specialized cargoes from the TGN 76. In stimulated neuroendocrine PC12 cells, neuropeptide hormones stored in SGs are released out of the cells when the SGs fuse with the plasma membrane. The number of SGs juxtaposed to the plasma membrane was lower in mitotic than in interphase cells 77, supporting the notion that TGN-to-plasma membrane trafficking is reduced in mitosis.

Contrary to this observation, glycosaminoglycan chains (GACs) synthesized in CHO cells were transported from the TGN to the plasma membrane at the same levels in interphase and mitotic cells 49. These apparent contradictions in the data on TGN-to-cell surface transport suggest that during mitosis, differential regulation of distinct cargoes in specialized transport vesicles could exist. Alternatively, cargoes secreted in a regulated manner in SGs might be affected to a different extent by the mitotic phase as compared to constitutively exported cargoes such as GACs. Also, it could well be that differences exist between the cell lines examined and since SGs are found in specialized cells, data might not be representative of all transport carriers.

## Disruption to the structural organization of various secretory organelles during mitosis could explain the down-regulation of protein trafficking

### Alterations in RER structure could prevent cargo export

Given that the mammalian cells undergo an open mitosis, the disruption of the protein transport might be a consequence of the gross structural reorganization of the RER. During interphase, the RER in mammalian cells is continuous with the nuclear envelope and extends to the rest of the cell as peripheral tubules and sheets 78. In an open mitosis, the nuclear envelope breaks down in early mitosis 79, with the nuclear envelope retracting into the RER and the RER undergoing reorganization.

There are disagreements over how the structure of the RER changes during mitosis. In HeLa cells, the mitotic RER assumes an extended cisternal arrangement, with a few tubular structures juxtaposed to the mitotic spindles 80. However, it was also reported that the RER reorganizes from sheets to tubules in mitotic CHO and HeLa cells 81, 82. The differences reported could arise from technical variations in the processing and analyses of the samples.

Interestingly, *Saccharomyces cerevisiae* goes through a closed mitosis with an intact nuclear envelope 79 and an absence of gross RER reorganization 83. In the yeast cells, constitutive export of proteins such as invertase and α-factor occurs at all stages of the cell division cycle 84, 85. Although this supports the idea that continued protein trafficking could occur in cells with closed mitosis where the RER structure is maintained, there is as yet no evidence showing that the reorganization of the gross RER structure in mammalian cells per se directly leads to a reduction in protein trafficking.

### Mitotic CDK1 activity promotes fragmentation of the Golgi apparatus

Similar to the RER, the Golgi apparatus is also altered structurally in mitosis. During interphase, the maintenance of the Golgi structure depends upon both the arrival of COPII vesicles from the RER fusing with the Golgi as well as the COPI vesicles that recycle membranes and proteins during intra-Golgi transport 68, 86. In mitosis, Golgi fragmentation could result from the reduced number of ERES and pre-budding complexes at the RER induced by the mitotic CDK1 activity (see above).

In addition, a tethering factor associated with the Golgi membrane known as GM130 is phosphorylated 87 by the mitotic CDK1 at Ser25 88. This prevents the binding between GM130 on the Golgi membrane and p115, which associates with a tethering factor Giantin on COPI vesicles 31. Due to the loss of binding between GM130 and p115 in mitosis 87, 88, COPI vesicle tethering to the Golgi cisternae is disrupted, which eventually leads to Golgi fragmentation 68, 71.

Other targets of the mitotic CDK1 include the Golgi reassembly stacking proteins (GRASPs), GRASP55 and GRASP65 72. As part of the coiled-coil class of tether proteins, the highly conserved GRASP55 and GRASP65 play a role in tethering of Golgi membranes needed to form Golgi cisternal stacks and ribbons. Phosphorylation of several serine/threonine residues at the C-terminal ends of the GRASP proteins by the mitotic cyclin B1- and cyclin B2-CDK1 complexes 89–91 disrupts the trans-oligomerization in GRASP proteins. This causes the Golgi to break up 91, 92, further highlighting the effect of the mitotic CDK1 on Golgi structure.

The structural organization of the Golgi further depends upon the AAA ATPase, p97 together with its associated proteins, p47 57 and p37 93, to mediate membrane fusion during Golgi reassembly. Phosphorylation of p47 at Ser140 by the mitotic CDK1 inhibits binding of p47 to the Golgi 94. Likewise, p37 phosphorylated at Ser56 and Thr59 by the mitotic CDK1 failed to bind Golgi membranes 95. The loss of association of p47 or p37 with Golgi membranes diminishes p97's ability to promote membrane fusion and hence maintain the Golgi structure in mitosis.

While the mitotic CDK1 induces Golgi disassembly, the significance of Golgi fragmentation in suspending protein trafficking has not been fully investigated. In fact, the finding that breaking down the Golgi apparatus by the small interfering RNA (siRNA)-mediated knock-down of *GM130* in HeLa cells did not affect VSV-G trafficking 96 implies that fragmentation of the Golgi apparatus perhaps has no influence on intra-Golgi transport. It should be noted though that the experiments were performed in interphase cells, and as such, might not completely reflect the condition in mitotic cells. It might be possible to test the effect of Golgi fragmentation on protein trafficking by permeabilizing *GM130* siRNA cells with streptolysin-O and incubating the permeabilized cells with or without mitotic lysates and examining the transport of VSV-G.

### Structural changes in cytoskeleton could stop vesicular movements

Cytoskeletal tracks upon which the secretory vesicles travel are highly altered in mitosis. The microtubules undergo dynamic organization not only in interphase cells 97, but are also reorganized to form spindles needed to segregate chromosomes in mitosis 98. Actin filaments that contribute to the delivery of secretory vesicles to the plasma membrane also undergo significant re-structuring as cells enter mitosis 99.

The maintenance of the Golgi structure depends upon both microtubules and actin 41, 97. The various functions of the microtubules and actin filaments have significant implications in the down-regulation of protein trafficking during mitosis. Understanding the contribution of the mitotic CDK1 activity in the gross structural changes of the cytoskeleton (e.g. 100, 101) might shed more light on how cells coordinate intracellular protein transport with cytoskeletal elements in mitotic cells and perhaps in interphase cells where microtubules or actin filaments might also undergo reorganization.

## Protein trafficking is re-established by Myt1 and Wee1 kinases during exit from mitosis

Exit from mitosis, characterized by a reduction in the mitotic kinase activity, is largely due to the destruction of the mitotic cyclins 102. In addition, the phosphorylation of CDK1 at the conserved Thr14 and Tyr15 residues by Myt1 and Wee1 kinases causes inactivation of the mitotic CDK1 activity 103 at the end of mitosis 104, 105.

Recently, it was shown that the down-regulation of Myt1 late in mitosis led to abnormal RER and Golgi morphologies in HeLa cells 105. However, alleviating the inhibition on the early secretory processes, by the destruction of the mitotic CDK1 activity, is insufficient for the re-formation of the RER 78, 80, 82 and Golgi 68 that presumably allows cargo transport through the Golgi 106. Rather, Myt1 appears to be needed for the re-formation of the RER and Golgi apparatus in addition to its role in inactivating the mitotic CDK1. The targets for Myt1 and Wee1 kinases that lead to changes in RER and Golgi structures have yet to be identified.

## Protein trafficking during exit from mitosis is needed for cytokinesis

Another noteworthy issue is the effect of CDK1 activity on the movement of vesicles from the post-Golgi to the cleavage furrow at the cytokinesis site at the end of mitosis 107–109. In metaphase, presumably, the movement should be inhibited, as premature furrow ingression prior to proper nuclear division would be detrimental to the dividing cells. Subsequent to nuclear division, the movement of vesicles out of the post-Golgi to the cleavage furrow is required for cytokinesis.

In mammalian cells, endocytic markers have been observed to concentrate at the cleavage furrow, which is the site of cytokinesis towards the end of mitosis 110. The internalization of membranes and proteins at the cleavage furrow helps shape the plasma membrane and promotes membrane scission that separates the dividing cells 111. This process is also under the tight control of cell cycle regulators 112.

## Future prospects

That the mitotic regulator CDK1 promotes not only events such as chromosome segregation but also down-regulates protein trafficking in mitosis highlights its central role in coordinating diverse processes in the cell. This is critical for ensuring proper progression through mitosis to produce viable progeny cells. Specific details as to how the CDK1 might affect the different stages of the secretory pathway warrant further studies using synchronized cells as well as in vitro assays to provide a more complete description of how the protein trafficking process is regulated in mitosis and also in interphase. In addition to endogenous cargoes, exogenously expressed cargoes, such as green fluorescent protein (GFP) fusions, could be used to dissect how the interactions between the CDK1 and protein trafficking components lead to dynamic changes in protein transport in live cells, which is not possible when examining endogenously expressed proteins.

Currently, several key questions about protein trafficking in mitosis remain unanswered, such as how the COPII coats are affected by the mitotic CDK1. Studies using in vitro RER budding assays (e.g. 113) or permeabilized cells in G1 (e.g. 55) in the presence or absence of the CDK1 complex could be performed to determine the effects of the mitotic CDK1 activity on COPII coat assembly on RER membranes. Moreover, predicted CDK1 phosphorylation motifs in COPII coat proteins could be mutated to ascertain if indeed the COPII coats are directly modified by CDK1 and whether the modifications are functionally relevant. Likewise, how the assembly of the ERES is inhibited by the mitotic CDK1 activity studied using similar set-ups could serve as an important starting point to examine protein export during mitosis. From such investigations, the molecular details of ERES assembly could emerge, which might also be relevant in interphase cells.

The effect of alterations in RER structure per se on protein export needs to be verified. For instance, structural reorganization of the RER in G1 cells devoid of CDK1 activity could be induced by altering the levels of proteins such as reticulons that modulate RER structure 114, and the effect on COPII coat recruitment or ERES assembly could be observed using various techniques such as fluorescence microscopy. Whether mitotic CDK1 activity inhibits protein export, independently of its role in promoting structural changes in the RER, could be investigated using G1 cells with altered reticulon function transfected with inducible constructs of CDK1 and cyclin B1. In the same line, G1 cells could be depleted of GM130 or GRASP proteins and examined to determine whether the budding of transport vesicles or trafficking of specific cargoes from the fragmented Golgi is affected by Golgi structure. Similarly, cells transfected with inducible constructs of CDK1 and cyclin B1 could be used to determine whether, besides promoting Golgi fragmentation, CDK1 activity inhibits protein trafficking.

The effects of the mitotic CDK1 activity on the reorganization of cytoskeletal structures 99, 100 needed for protein trafficking is a complex issue that might not be easy to resolve. This is because the cytoskeletal structures play roles not only in the organization of the RER and Golgi, but also in the biogenesis of transport vesicles and movement of vesicles. Nevertheless, it could be informative to perform mutational analysis of actin or microtubules (e.g. 100) or their associated proteins to look at possible effects on vesicle transport in motility assays in vitro.

It would also be relevant to study whether the inhibition of the secretory pathway at the end of mitosis is due merely to relieving the inhibitory effects of the mitotic CDK1 activity, as other kinases such as Wee1 and Myt1 are needed to re-establish the RER and Golgi 105. In this respect the identification of targets of these kinases could provide fresh ideas on how the RER and Golgi are reassembled to restart protein transport during mitotic exit.

Finally, using high-content screening methods incorporating GFP-fused cargoes, it might be possible to screen substrates of CDK1 115 for those that affect the protein transport process. This may also uncover novel components of the secretory pathway. I anticipate that the study of protein secretion in the context of mitosis would undoubtedly broaden our current understanding of how cells ensure timely delivery of proteins to the correct subcellular location.

## Abbreviations

CDK: cyclin-dependent kinase
CHO cells: Chinese hamster ovary cells
COPI/II: coatomer protein complex I/II
ER: endoplasmic reticulum
ERES: ER exit sites
ERGIC: ER-to-Golgi intermediate compartment
G1: gap phase 1
GEF: guanine nucleotide exchange factor
RER: rough ER
SG: secretory granule
VSV-G: G protein of the vesicular stomatitis virus
